# Turn-off colorimetric sensor for sequence-specific recognition of single-stranded DNA based upon Y-shaped DNA structure

**DOI:** 10.1038/s41598-018-30529-z

**Published:** 2018-08-13

**Authors:** Hong Zhang, Xintong Li, Fan He, Mingqin Zhao, Liansheng Ling

**Affiliations:** 1 College of Tobacco Science, Henan Agricultural University, Zhengzhou, 450002 P. R. China; 2 School of Chemistry, Sun Yat-Sen University, Guangzhou, 510275 P. R. China

## Abstract

A novel turn-off colorimetric sensor for sequence-specific recognition of single-stranded DNA (ssDNA) was established by combining Y-shaped DNA duplex and G-quadruplex-hemin DNAzyme. A G-rich single-stranded DNA (Oligo-1) displays peroxidase mimicking catalytic activity due to the specific binding with hemin in the presence of K^+^, which was able to catalyze the oxidation of colorless 2,2′-azinobis(3-ethylbenzothiazoline)-6-sulfonic acid (ABTS^2−^) by H_2_O_2_ to generate green ABTS•^−^ radical for colorimetric assay. Oligonucleotide 2 (Oligo-2) was partly complementary with Oligo-1 and the target DNA. Upon addition of target DNA, Oligo-1, Oligo-2 and target DNA can hybridize with each other to form Y-shaped DNA duplex. The DNAzyme sequence of Oligo-1 was partly caged into Y-shaped DNA duplex, resulting in the inactivation of the DNAzyme and a sharp decrease of the absorbance of the oxidation product of ABTS^2−^. Under the optimum condition, the absorbance decreased linearly with the concentration of target DNA over the range of 1.0–250 nM and the detection limit was 0.95 nM (3σ/slope) Moreover, satisfied result was obtained for the discrimination of single-base or two-base mismatched DNA.

## Introduction

Single nucleotide polymorphisms (SNPs) are single nucleotide variations that may be one-base substituted, deleted or inserted in a natural DNA sequence. SNPs make up a high proportion of human sequence variations and occur at about one per 500–1000 base pair in human genome^1^. In addition, numerous human diseases are associated with SNPs, such as cystic fibrosis, diabetes, sickle cell anemia, Alzheimer’s, mental illness and certain cancers^2^. Therefore, the discrimination of SNPs has attracted much attention for its importance in disease diagnosis, biomedical studies, food safety and environmental analysis. A series of sensors have been established for the recognition of SNPs. For example, SNPs are able to be discerned by elevating temperature with strict temperature control or lowering the salt concentration of the buffer^3,4^ for the small difference of thermal stability between single-base mismatched and perfect matched DNA under the conditions. Protein enzymes also have been used to distinguish SNPs. Restriction enzymes are also suitable to discriminate SNPs^5,6^, but it is only applied to the sequence containing enzyme recognition domain, which limit its application. Ligase can be applied to detect SNPs for any random DNA sequence^7–9^, but ligase is relatively expensive, vulnerable to the surrounding and also accompanying by nonspecific absorption phenomenon^10^. In view of the limitations of the existing methods, recent emergency of Y-shaped DNA duplex might provide potential application for the discrimination of SNPs.

Y-shaped DNA duplex consists of three complementary oligonucleotide components, two probes and a target DNA^11,12^. Two probes do not hybridize with each other without target DNA, but the hybridization occurs in the presence of target DNA through the formation of Y-shaped DNA duplex. This structure is more sensible to single-base mismatch even under mild conditions since a relatively short duplex is formed between probes and target DNA^13,14^. Therefore, the selectivity of Y-shaped DNA duplex is better than that of linear DNA duplex, which has widely used for the recognition of single-base mutation^15^.

DNAzyme is artificial single-stranded DNA with a specific sequence and can specifically catalyze some chemical and biological reaction. Recently, DNAzyme has attracted more and more attention owing to its merits of high thermal stability, low cost, ease of preparation and purification as compared with natural protein enzymes^16,17^. G-quadruplex-based DNAzyme that has peroxidase mimicking catalytic activity can catalyze the oxidation of 2,2′-azinobis(3-ethylbenzothiazoline)−6-sulfonic acid (ABTS^2−^), luminol, 4-chloro-1-naphthol (4-CN) or scopoletin (Sc) by H_2_O_2_ for the detection of colorimetric, chemiluminescence, electrochemical or fluorescence analysis^18–21^. Meanwhile, colorimetric sensor possesses significant advantages of it including simplicity, fast analysis, low cost and good sensitivity^22,23^. Therefore, considering the advantages of colorimetric sensors and the catalytic effect of G-quadruplex-based DNAzyme, a series of sensors were constructed for the detection of metal ions^24^, nucleic acid^25,26^, protein^27,28^ and small molecular^29,30^.

Herein, we developed a turn-off colorimetric sensor for the recognition of single nucleotide polymorphisms based upon Y-shaped DNA duplex and G-quadruplex-hemin DNAzyme. In the absence of target DNA, a G-rich single-stranded DNA (Oligo-1) did not hybridize with assistant DNA (Oligo-2), which induced the formation of DNAzyme in the presence of hemin and K^+^. When the probes were changed with target DNA (Oligo-3), Oligo-1, Oligo-2 and target DNA hybridized with each other to form Y-shaped DNA duplex, resulting in a sharp decrease of the absorbance of UV-Vis absorption spectrometry. The detection of single-stranded DNA can be realized by observing the optical signal change before and after the addition of target molecules. The assay could not only enhance the sensitivity for the catalytic activity of G-quadruplex-hemin DNAzyme, but also improve the selectivity for the structure of Y-shaped DNA duplex.

## Materials and Methods

### Materials

Dimethyl sulfoxide (DMSO) and hydrogen peroxide (30%) were purchased from Guangzhou Chemical Reagent Factory (Guangzhou, China). 3,6-dimethyl-2-(4-dimethylaminophenyl) benzo-thiazolium cation (Thioflavin T, ThT) and SYBR Green I (SGI) were purchased from Sangon Biotech Inc. (Shanghai, China). All oligonucleotides were obtained from Sangon Biotech Inc. (Shanghai, China) and the sequences were listed in Table 1. The stock solution of oligonucleotides was prepared with deionized water. The concentration of oligonucleotides was accurately quantified by UV-Vis absorption spectroscopy according to the extinction coefficients (ε_260nm_, M^−1^cm^−1^): A = 15400, G = 11500, T = 8700, C = 7400. Hemin was obtained from Aladdin Chemistry Co. Ltd. (Shanghai, China). The stock solution of 2.0 mM hemin was prepared in DMSO and stored in darkness at −20 °C. Tris-HCl buffer (pH 7.0, 150 mM KCl) was used in our experiments. All chemicals were used as received without further purification.

**Table 1:** Sequence of oligonucleotides.

Name	Sequence
Oligo-1 (Signal probe)^a^	5′-*CTA GTC AGT GTG GA***T GGG TTG GG**C GGG ATG GG−3′
Oligo-2 (Assistant probe)^b^	5′-**CCC AAC CCA** AAA TCT CTA GCC AG−3′
Oligo-3 (Target DNA)	5′-CTG GCT AGA GAT TTT CCA CAC TGA CTA G-3′
Oligo-4^c^	5′-CTG GCT AGA GAT TTT CCA CAT TGA CTA G-3′
Oligo-5^d^	5′-CTG GCT AGA GAT TTA CCA CAC CGA CTA G-3′

^a^The underlined bases are a G-rich sequence, the italic bases and the bold letters are duplex-forming sequence that are complementary with part of the target-DNA and Oligo-2, respectively. ^b^The bold letters and the underlined bases are complementary with Oligo-1 and the target DNA, respectively. ^c,d^The underlined letter identify the mismatched base.

### Apparatus

Centrifugation experiments were performed on an Anke GL-20G-II centrifuge (Anting Scientific Instrument Factory, China). The absorption spectra of radical anion ABTS•^−^ was carried on a TU-1901 double-beam spectro-photometer (Beijing Purkingje General Instrument Co. Ltd, China). A JASCO Model J-810-150S spectropolarimeter (JASCO International CO. Ltd, Japan) was used for the measurement of circular dicroism (CD) spectroscopy. Fluorescence spectra data were performed with an RF-5301PC spectrophotometer (Shimadzu, Japan).

### Colorimetric recognition of target single stranded DNA

250 μL of Oligo-1 (1.5 μM), 250 μL of Oligo-2 (1.5 μM) and different concentration of target DNA (Oligo-3) were heated to 88 °C and held for 10 min, then gradually cooled down to room temperature to form Y-shaped DNA duplex. The mixture was incubated with 50 μL of hemin (15 μM) for 1 h at room temperature to allow formation of DNAzyme. Finally, 20 μL of ABTS^2−^ (40 mM) and 20 μL of H_2_O_2_ (25 mM) were added to above mixture to initiate the colorimetric reaction.

### Measurement of CD spectroscopy

450 μL of Tris-HCl (pH 7.0, 150 mM KCl) containing 5 μM Oligo-1, 5 μM Oligo-2 and 10 μM target DNA were heated to 88 °C and held for 10 min, then gradually cooled down to room temperature. Subsequently, 20 μL of hemin (200 μM) was added to the above mixture and allowed to incubate for 1 h at room temperature. Finally, the sample was transformed to 400 μL cuvette with 0.1 cm path length. The measurement was performed from 200 nm to 350 nm with a scanning rate of 10 nm/min, with a response time of 1 s and a band width of 1.71 nm. The reference solution was Tris-HCl (pH 7.0, 150 mM KCl) with hemin.

### Measurement of fluorescence spectra

250 μL of Oligo-1 (1.5 μM), 250 μL of Oligo-2 (1.5 μM) and 500 μL of Oligo-3 (0.4 μM) were heated to 88 °C and held for 10 min, then gradually cooled down to room temperature to form Y-shaped DNA duplex. Subsequently, 50 μL of ThT (10 μM) was added to the mixture and allowed to incubate for 1 h at room temperature. The measurements were recorded by fluorescence spectrophotometer with excitation at 425 nm and emission wavelength from 450 to 600 nm.

## Results and Discussion

### Scheme of the assay

**Figure 1:**
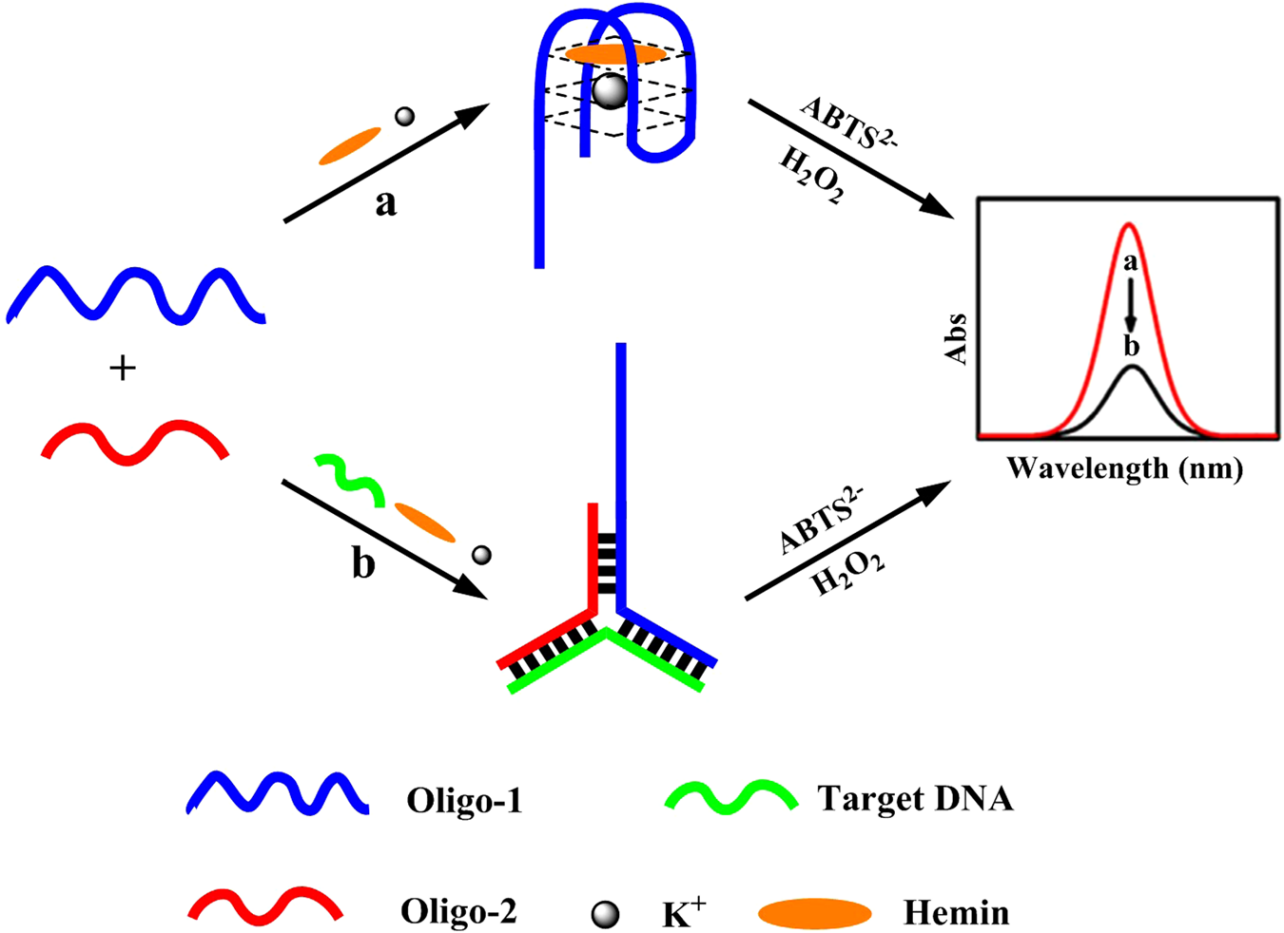
Schematic illustration for sequence-specific recognition of single-stranded DNA based upon Y-shaped DNA duplex and G-quadruplex-hemin DNAzyme.

The scheme of the assay is depicted in Fig. 1. Oligo-1 is signal probe that be composed of a G-quadruplex-hemin DNAzyme sequence and a recognition domain for part of the target DNA (Table 1). Oligo-2 is used as assistant probe, which is partially complementary with Oligo-1 and target DNA. In the absence of target DNA, Oligo-1 does not hybridize with Oligo-2, and the free Oligo-1 can catalyze the oxidation of colorless 2,2′-azinobis(3-ethylbenzothiazoline)−6-sulfonic acid (ABTS^2−^) by H_2_O_2_ into green ABTS•^−^ radical. However, Y-shaped structure formed between Oligo-1, Oligo-2 and target DNA upon addition of target DNA, resulting in the inactivation of the DNAzyme and a sharp decrease of the absorbance of UV-Vis absorption spectroscopy. The detection of target DNA can be realized by observing the optical signal change.

### Absorption spectrum

**Figure 2:**
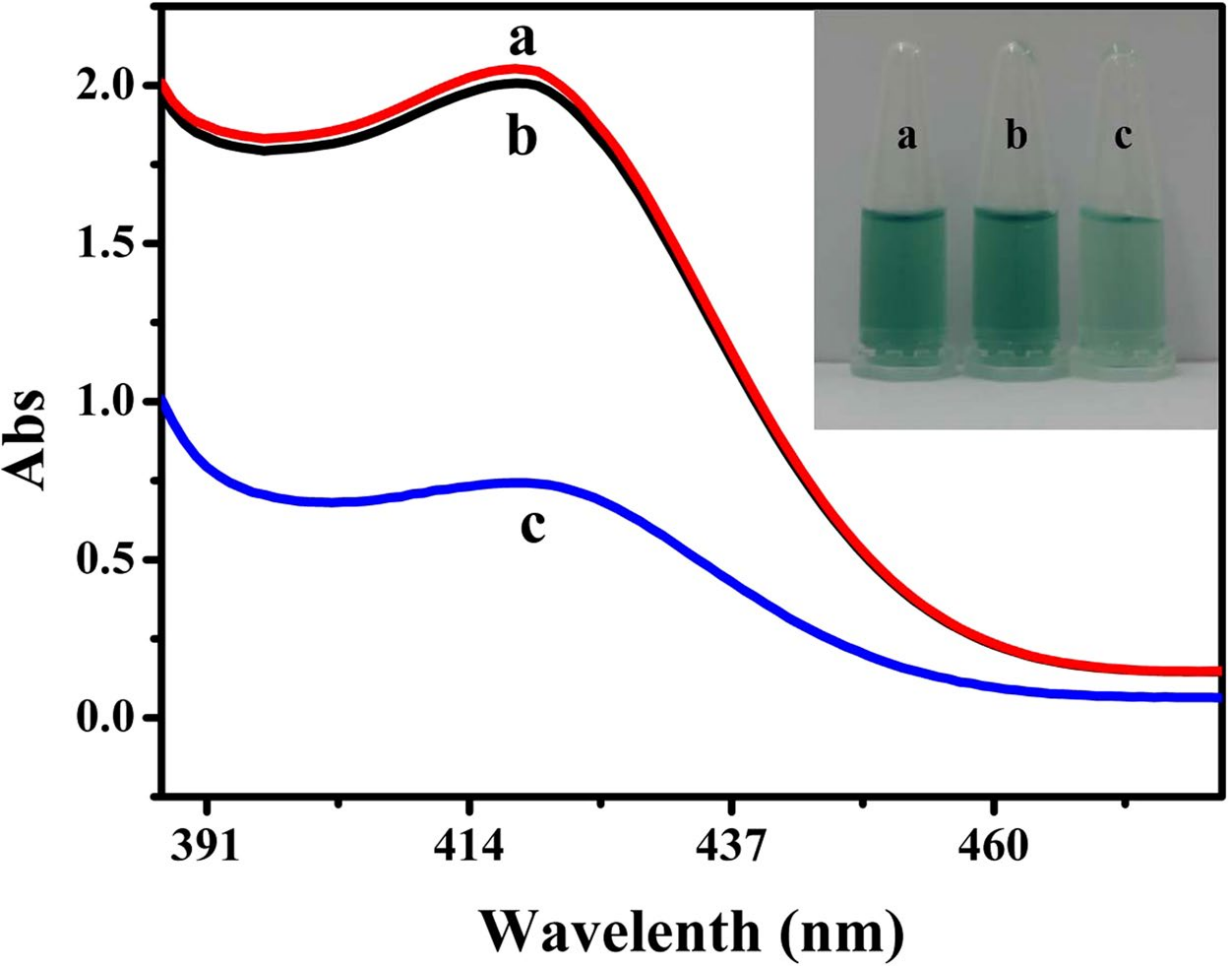
Typical photograph and absorption spectra of ABTS^2−^ and H_2_O_2_ mixture catalytically oxidized by G-quadruplex-hemin DNAzyme under different conditions. (a) Oligo-1; (b) a + Oligo-2; (c) b + 500 nM target DNA. Experimental conditions: 1.5 μM Oligo-1, 1.5 μM Oligo-2, 15 μM hemin, 40 mM ABTS^2−^ and 25 mM H_2_O_2_. 10 mM Tris-HCl buffer (pH 7.0, 150 mM KCl) was used.

To evaluate the possibility of the assay, UV-Vis absorption spectroscopy of the mixture of ABTS^2−^ and H_2_O_2_ was investigated under different conditions. As shown in Fig. 2, the absorbance of Oligo-1 was strong, which indicated that Oligo-1 can form DNAzyme and exhibited a very high catalytic activity towards H_2_O_2_−ABTS^2−^ system_._ There was almost no change after incubation with Oligo-2, which demonstrated that Oligo-1 could not hybridize with Oligo-2 solely. But the absorbance decreased dramatically with further addition of target DNA, which might be due to the change of the conformation of Oligo-1.

### Validation of the conformational switches

**Figure 3:**
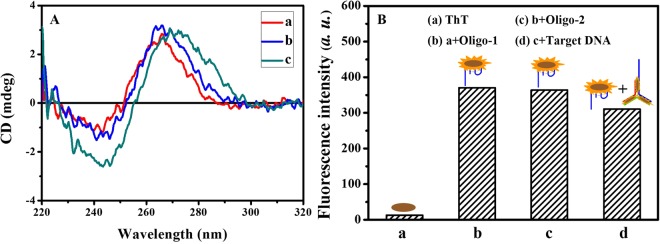
(**A**) CD spectroscopy of DNA molecule under different conditions. (a) Oligo-1; (b) a + Oligo-2; (c) b + target DNA. (**B**) Fluorescent validation of the formation of G-quadruplex through ThT fluorescence. (a) ThT; (b) a + Oligo-1; (c) b + Oligo-2; (d) c + target DNA.

To explore the conformational switches of the mentioned phenomena, circular dicroism (CD) spectroscopy of DNA was investigated under different conditions. As shown in Fig. 3A, a positive peak at 265 nm and a negative peak at around 245 nm could be observed in the CD spectroscopy of Oligo-1, indicating the formation of G-quadruplex. There was no obvious change upon addition of Oligo-2, which indicated that Oligo-1 still kept G-quadruplex structure. The spectroscopy appeared a positive peak at around 275 nm and a negative Cotton effect of DNA helicity at 245 nm with further addition of Oligo-3, which revealed that the conformation of Oligo-1 changed from G-quadruplex structure to duplex structure^31^.

ThT had been demonstrated as highly fluorescent responsive for G-quadruplexe compared with single/double-stranded^32–34^. Therefore, ThT was used to further observe the conformational switches. As shown in Fig. 3B, strong fluorescence was observed in the presence of Oligo-1, which indicated that Oligo-1 can form G-quadruplex and strongly bind with ThT to generate significantly enhanced fluorescent signal. Almost no remarkable change was observed after incubation with Oligo-2, which demonstrated that Oligo-1 could not hybridize with Oligo-2 and was able to fold into a G-quadruplex. But, the fluorescence intensity decreased dramatically with further addition of target DNA, accompany by a spontaneous conformational change from G-quadruplex to Y-shaped DNA duplex. These were corresponding exactly with the results of CD spectroscopy.

### Optimization of experimental conditions

The turn-off colorimetric detection of target DNA depended on the concentration of Oligo-1, Oligo-2, hemin, KCl, ABTS^2−^ and H_2_O_2_. These factors influenced the change of absorbance (ΔA), here we defined as ΔA = A_blank_-A (A_blank_ refer to the absorbance of ABTS^2−^ and H_2_O_2_ mixture in the presence of Oligo-1 and Oligo-2, A represented the absorbance of ABTS^2−^ and H_2_O_2_ mixture in the presence of Oligo-1, Oligo-2 and target DNA).

**Figure 4:**
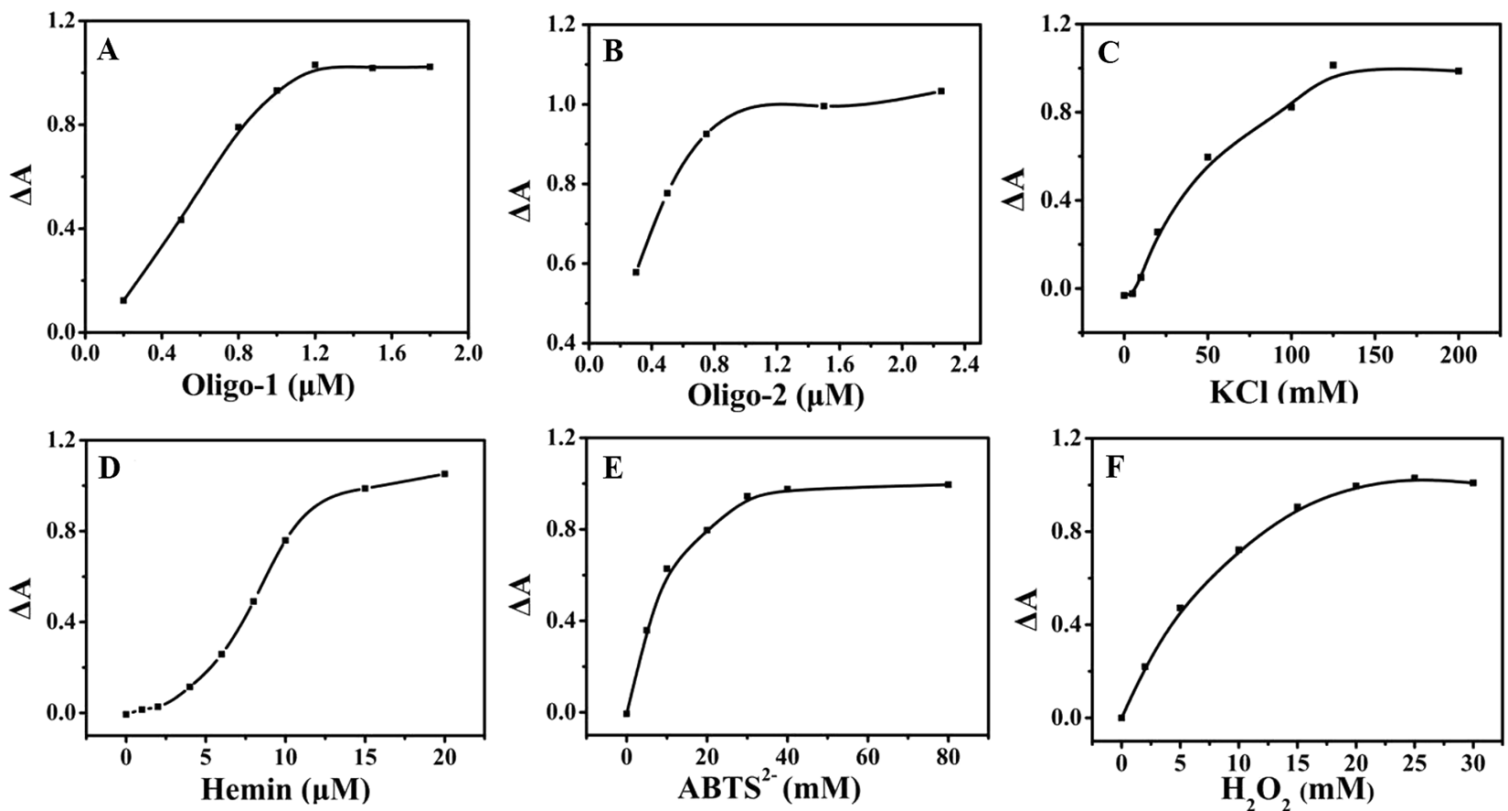
Effect of the sconcentration of (**A**) Oligo-1, (**B**) Oligo-2, (**C**) KCl, (**D**) hemin, (**E**) ABTS^2−^ and (**F**) H_2_O_2_ on ΔA.

The concentration of Oligo-1 decided the number of DNAzyme. Therefore, the effect of Oligo-1 concentration was investigated. As shown in Fig. 4A, ΔA increased rapidly with the increase of Oligo-1 concentration over the range from 0.2 μM to 1.2 μM and reached a plateau over the range from 1.2 μM to 1.8 μM, so 1.5 μM of Oligo-1 was used for the research.

Oligo-2 can repress the formation of G-quadruplex structure of Oligo-1 in the presence of target DNA, so the effect of Oligo-2 concentration was measured (Fig. 4B). ΔA increased gradually with the concentration of Oligo-2 over the range of 0.3–0.75 μM, ΔA reached maximum and kept a plateau if the concentration of Oligo-2 was higher than 1.0 μM. Therefore, 1.5 μM of Oligo-2 was selected in the assay.

A G-rich sequence can form G-quardruplex with K^+^, so the concentration of K^+^ was investigated. As shown in Fig. 4C, ΔA was found to be proportional to the KCl concentration over the range of 0–125 mM, then it reached a plateau when the concentration of KCl was higher than 125 mM. Therefore, 150 mM of KCl was selected for further research.

K^+^-stabilized G-quardruplex exhibited catalytic activity with hemin as the cofactor. Thereby, the concentration of hemin played an important role in the activity of DNAzyme. Herein, the effect of hemin concentration was investigated in Fig. 4D. ΔA increased rapidly with the increase of hemin concentration over the range of 0–12.0 μM, then it kept a plateau during the range of 12.0–20.0 μM. Therefore, 15.0 μM of hemin was selected for further research.

ABTS^2−^ and H_2_O_2_ were two reactants for the colorimetric reaction, which monitored the colorimetric process. Thus, the effect of the concentration of ABTS^2−^ and H_2_O_2_ were investigated. As shown in Fig. 4E, the effect of the concentration of ABTS^2−^ was studied over the range from 0 to 80 mM. ΔA increased dramatically with the increase of ABTS^2−^ concentration during the range of 0–30 mM, then it reached maximum and kept at a plateau when the concentration of ABTS^2−^ was higher than 30 mM. Thus 40 mM ABTS^2−^ was selected for the research. As shown in Fig. 4F, ΔA increased with the increase of H_2_O_2_ concentration during the range of 0–20 mM, and then it kept at a plateau when the concentration varied from 20 mM to 30 mM. Thereby, 25 mM of H_2_O_2_ was selected for the assay.

### Effect of different assistant probe

**Figure 5:**
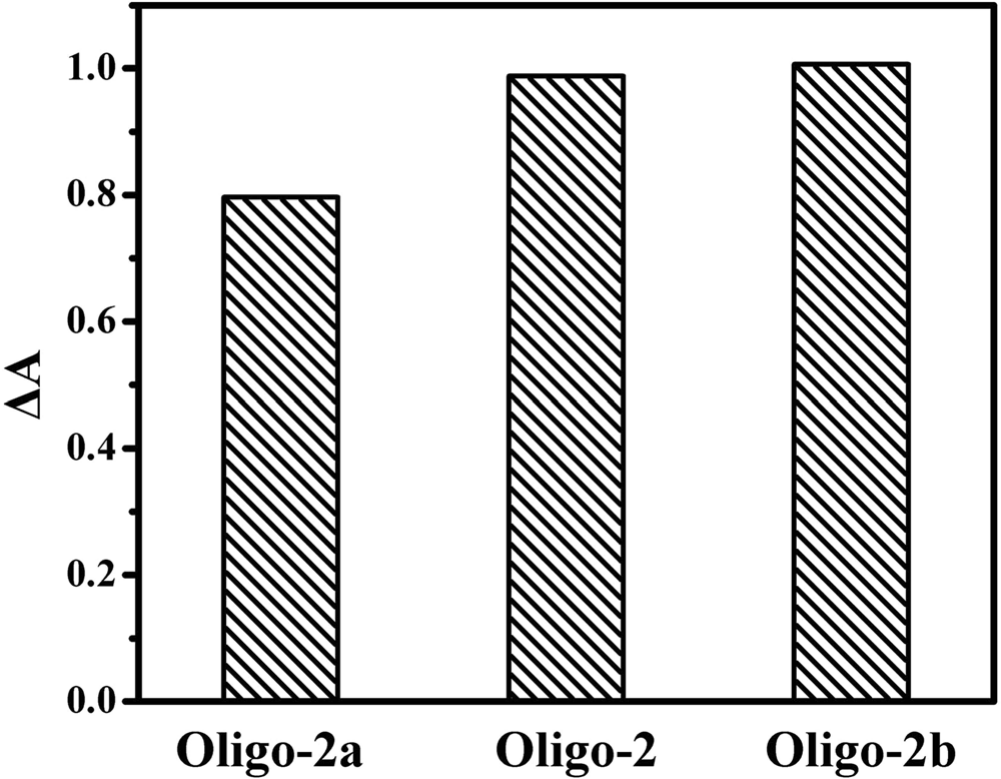
Effect of different sequence of assistant probe on ΔA. Experimental conditions: 1.5 μM signal probe, 150 mM KCl, 15 μM hemin, 40 mM ABTS^2−^, 25 mM H_2_O_2_ and1.5 μM assistant probe. The concentration of Oligo-3 was 200 nM. 10 mM Tris-HCl buffer (pH 7.0, 150 mM KCl) was used.

Signal probe and assistant probe were both comprised of duplex-forming sequence and can anneal to each other in the presence of target DNA. The number of base pairs between signal probe and assistant probe controlled the sensitivity of the assay. If the complementary segment was long enough, signal probe could hybridize with assistant probe in the absence of target DNA, resulting in the high background. However, if the complementary segment was too short, the formed Y-shaped DNA duplex was unstable even with target DNA. Therefore, oligonucleotides 5′-CCA ACC CA AAA TCT CTA GCC AG-3′ (Oligo-2a), 5′-CCC AAC CCA AAA TCT CTA GCC AG-3′ (Oligo-2) and 5′-G CCC AAC CCA AAA TCT CTA GCC AG-3′ (Oligo-2b) were used to act as assistant probe, which could hybridize with signal probe with 8, 9 and 10 base pairs, respectively. As shown in Fig. 5, the change of absorbance (ΔA) was almost the same upon addition of Oligo-2 and Oligo-2b, which was higher than that of Oligo-2a. Therefore, Oligo-2 was used in later experiment.

### Sensitivity of the assay

**Figure 6:**
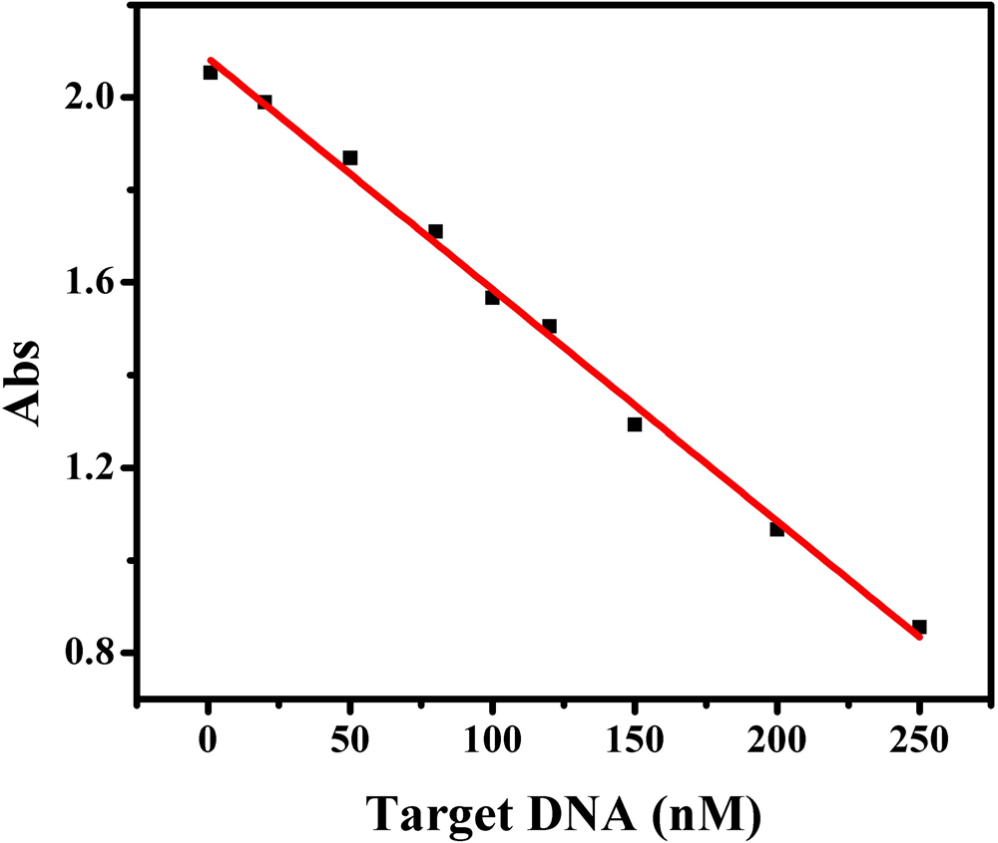
Calibration curve of the assay. Experimental conditions: 1.5 μM signal probe, 1.5 μM assisstant probe, 15 μM hemin, 40 mM ABTS^2−^ and 25 mM H_2_O_2_. 10 mM Tris-HCl buffer (pH 7.0, 150 mM KCl) was used in the experiment.

Under the optimum conditions, the relationship between the concentration of target DNA and absorbance was evaluated. As demonstrated in Fig. 6, the absorbance decreased linearly with the increase of the concentration of target DNA during the range of 1.0–250 nM. The linear regression equation was Y = −0.0050 C + 2.1 (C in nM, R = 0.9975) with the detection limit (DL) of 0.95 nM, which was obtained from the equation DL = 3σ/slope.

### Selectivity of the assay

**Figure 7:**
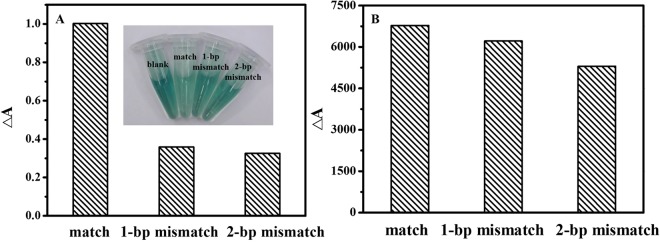
Sequence selectivity of the assay toward (**A**) Y-shaped DNA duplex and (**B**) linear DNA duplex. Experimental conditions: 1.5 μM Oligo-1, 1.5 μM Oligo-2, 15 μM hemin, 40 mM ABTS^2−^ and 25 mM H_2_O_2_. The concentration of Oligo-3, Oligo-4, Oligo-5 and block DNA was 200 nM. 10 mM Tris-HCl buffer (pH 7.0, 150 mM KCl) was used.

Sequence selectivity was important to the assay, two control sequences containing 1-bp mismatch (Oligo-4) and 2-bp mismatch (Oligo-5) were designed to evaluate the selectivity of the assay. As shown in Fig. 7A, the change of absorbance (ΔA) of 1-bp mismatch and 2-bp mismatched DNA were lower than that of target DNA. It indicated that single base mismatched sequence could be discriminated well from the target DNA.

To verify the advantage for discrimination of single-base mutation by using Y-shaped DNA duplex, linear DNA duplex acted as the control DNA structure was designed for the fluorescent detection of single-stranded DNA by using the double-stranded DNA-binding dye SYBR Green I (SGI). Block DNA (5′-CTA GTC AGT GTG GAA AAT CTC TAG CCA G-3′) acted as the recognition probe was complementary with the target DNA (Oligo-3). As shown in Fig. 7B, the value of ΔA for one base and two bases mutated sequences were 93.10% and 81.85% of that for perfect target DNA by using linear DNA duplex, which showed tiny change as compared to that of perfect target DNA. Meanwhile, ΔA of the DNA mismatched by one base and two bases were 35.80% and 32.55% of that for perfect target DNA by using Y-shaped DNA duplex, respectively (Fig. 7A). These results indicated that the selectivity of Y-shaped DNA duplex is better than that of linear DNA duplex.

## Conclusions

A novel turn-off colorimetric sensor for the recognition of single-stranded DNA was established by combining Y-shaped DNA duplex and G-quadruplex-hemin DNAzyme. The assay had high sensitivity and selectivity for the use of G-quadruplex-hemin DNAzyme and Y-shaped DNA duplex structure. Moreover, no expensive and sophisticated instruments and no tedious DNA covalent labelling procedure was used in the assay, which reduce cost of the assay. Under the optimum conditions, the proposed sensor allowed the detection of target DNA over the range of 1.0–250 nM with a detection limit of 0.95 nM. Furthermore, single-base and two-base mismatched sequence could be discriminated well from the target DNA.
